# Supplementary material for: nucMACC: An MNase-seq pipeline to identify structurally altered nucleosomes in the genome
Source: Sci Adv. 2024 Jul 3;10(27):eadm9740. doi: 10.1126/sciadv.adm9740 (PMC11221511; doi:10.1126/sciadv.adm9740)
Supplement: Supplementary file 1 — Supplementary Text Figs. S1 to S8 Legends for data S1 to S4 [file sciadv.adm9740_sm.pdf]

Science Advances
AAAS

Supplementary Materials for

nucMACC: An MNase-seq pipeline to identify structurally altered nucleosomes in the genome

Sara Wernig-Zorc *et al.*

Corresponding author: Uwe Schwartz, uwe.schwartz@ur.de; Gernot Längst, gernot.laengst@ur.de

Sci. Adv. **10**, eadm9740 (2024)
DOI: 10.1126/sciadv.adm9740

The PDF file includes:

Supplementary Text
Figs. S1 to S8
Legends for data S1 to S4

Other Supplementary Material for this manuscript includes the following:

Data S1 to S4

Supplementary Text

The nucMACC pipeline

The nucMACC pipeline requires a minimum of two MNase-seq titration conditions as input, preferentially in combination with a histone immunoprecipitation step, to exclude non-histone protein-mediated DNA-binding events. Starting from raw sequencing files in the FASTQ format, the nucMACC pipeline first assesses the sequencing quality. Next, the reads are aligned to the provided reference genome and subsequently mapped reads are filtered based on mapping quality (Figure S1). Optionally ambiguous genomic elements, such as blacklisted regions or mitochondrial chromosomes, can be removed from the analysis. To obtain high-resolution nucleosome positions, fragments between 140 and 200 bp in length, the typical size of mono-nucleosomal DNA, are selected (Figure 1B). Several quality checks including fragment size profiles, fragment statistics and TSS plots, are performed to ensure the integrity and reliability of the data analysis process. When replicates are available the pipeline provides a separate workflow MNaseQC to check the quality and consistency between replicates. In MNaseQC statistics are reported for individual replicates and a principal component and a correlation analysis are additionally performed. Based on the quality, appropriate samples can be selected and an additional entry point in the nucMACC pipeline provides seamless integration with subsequent analysis. Next, size-selected fragments of all MNase titration conditions are pooled and used to derive a comprehensive map of the genome-wide nucleosome positions.

Recently, a metric to measure chromatin accessibility, termed MACC (MNase accessibility), was introduced (*31*). As the MACC scores provide an elegant way to measure chromatin accessibility based on MNase-seq data, we adapted the MACC scoring system but changed the principle of calculation to obtain a higher annotation resolution and accuracy. Instead of counting the MNase reads in arbitrary genomic bins as in the original MACC version, we quantify the MNase accessibility directly at the defined nucleosome positions, which results in a specific accessibility score for each individual nucleosome (nucMACC). Next, linear regression is conducted on the normalized fragment frequencies for each nucleosome (Figure S1, see Methods section for details). The slope of the regression line multiplied by minus one is used as the nucMACC score. Since MNase exhibits an increasing AT preference at higher concentrations, raw nucMACC scores are normalized for the underlying GC content using a LOESS regression (*30*).

As a new feature of the nucMACC pipeline fragments shorter than 140 bp, representing sub-nucleosomal DNA fragments, are used to measure nucleosome stability. We use only the lowest MNase concentration to call these sub-nucleosomal positions (called sub-nucleosomes in the remaining text), as at higher MNase titration conditions, these unstable nucleosomes are fully hydrolyzed (Figure 1A, blue nucleosome). Analogous to the nucMACC scores, sub-nucleosomal fragment frequencies for each MNase titration condition are quantified at the defined sub-nucleosome positions. The slope of the regression is used to measure nucleosome stability and normalized to the underlying GC content. The normalized score is referred to as the sub-nucleosome MNase accessibility score (sub-nucMACC).

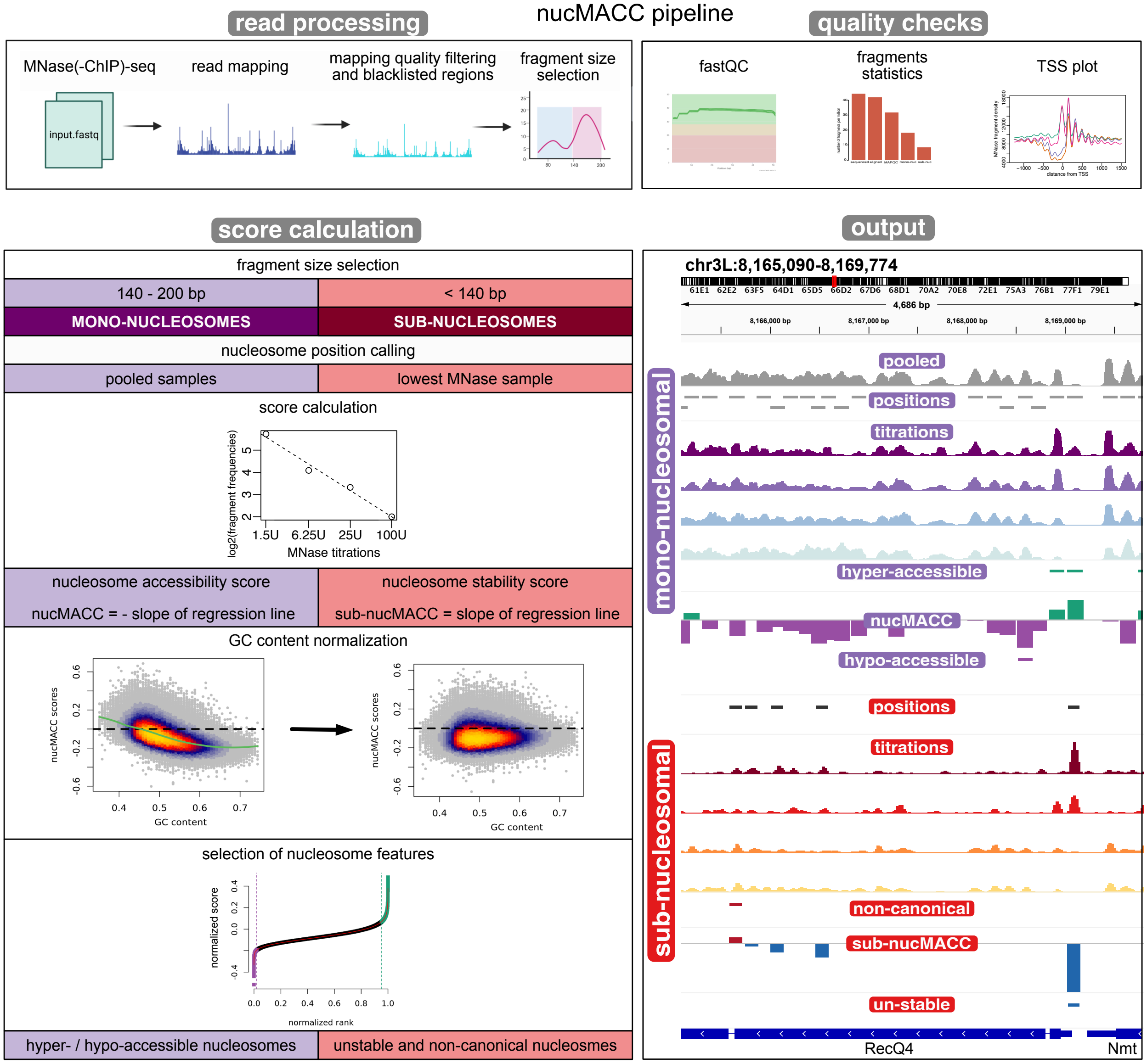

Fig. S1.

Overview of nucMACC pipeline. The nucMACC pipeline starts with raw data in fastq format and requires a minimum of two MNase conditions. Fragments are mapped to the reference genome and aligned fragments are filtered based on mapping quality. In the next step, fragments are divided into sub-nucleosomal and mono-nucleosomal-sized fragments, which are processed separately (left top panel). Quality reports are generated at different steps of the pipeline to control each step of the analysis and the underlying data (right top panel). For nucleosome position calling all MNase concentration conditions are pooled, whereas for sub-nucleosomes, only the lowest MNase titration is used to call sub-nucleosomal positions. Sub-nucleosomal positions are consequently filtered by previously called mono-nucleosomal positions to obtain enriched sub-nucleosome positions. Then a nucMACC score is calculated by counting fragments per nucleosome position in each MNase condition. The slope of a linear regression fit is determined and represents the raw score, which is normalized to the underlying GC% content in the following step. The normalized

score is referred to as the nucleosome MNase accessibility score (nucMACC) or sub-nucMACC score, respectively. In the final step, special nucleosome groups are obtained by analyzing where the (sub-)nucMACC score considerably deviates from the mean. From the mono-nucleosome fraction we obtain stable, hypo, and hyper-accessible nucleosomes. While the special nucleosomes from the sub-nucleosomal fraction represent un-stable and non-canonical nucleosomes (left bottom panel). The pipeline generates several output files, such as nucleosome positions, MNase profiles, (sub-)nucMACC scores, or special nucleosome features, which can be directly visualized in the genome browser (right bottom panel).

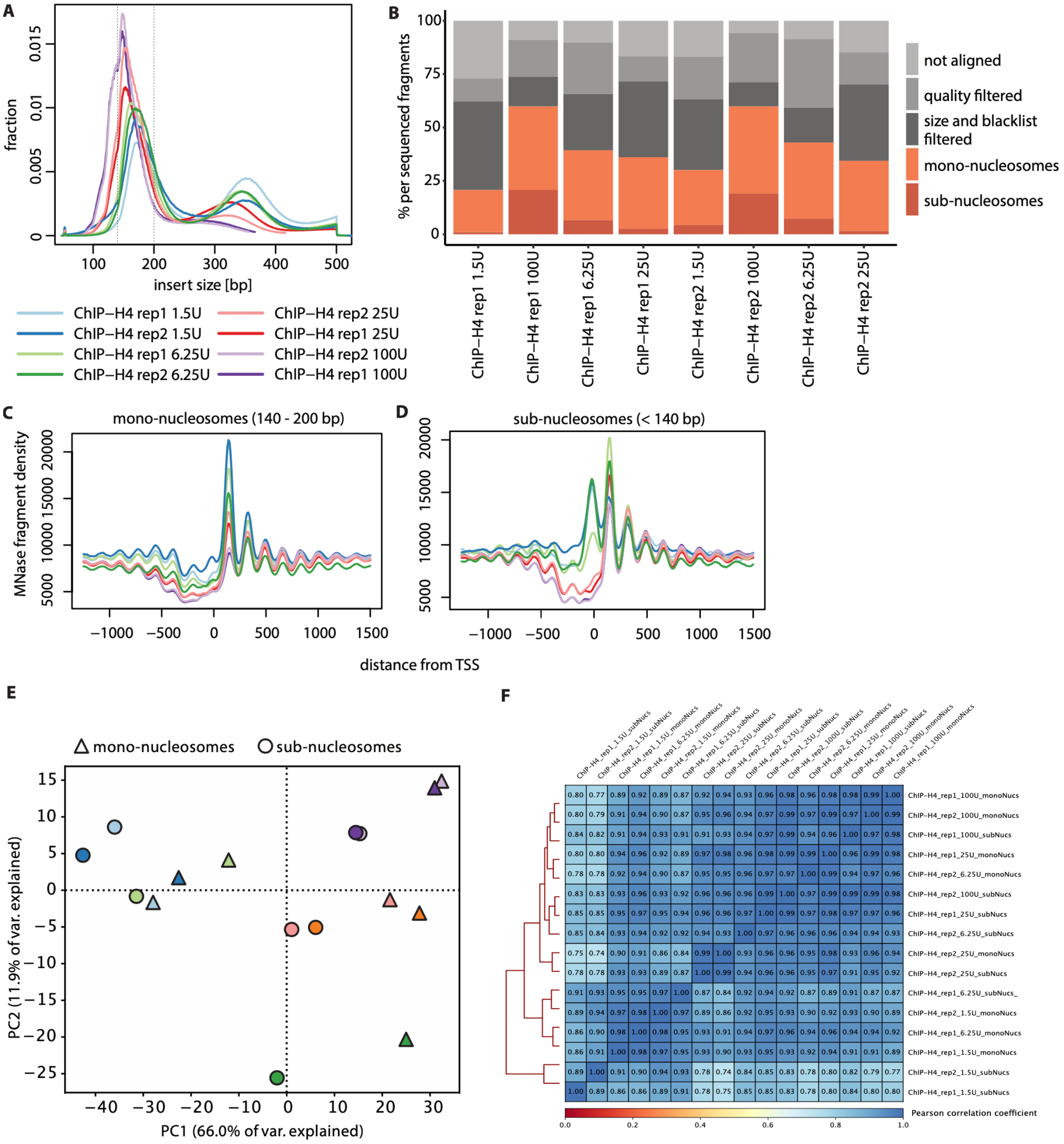

Fig. S2.

Replicate consistency check using MNaseQC workflow. (**A**) Fragment size distribution of H4-ChIP MNase titrations in *D. melanogaster* (*31*). Dashed lines denote the fragment size cutoffs used for mono- (140 – 200 bp) and sub-nucleosome (< 140 bp) analysis. (**B**) Fragment statistics for read processing steps including alignment, MAPQ filtering post alignment, size-selection (mono-nucleosomes 140-200 bp and sub-nucleosomes < 140 bp) and blacklisted region filtering. (**C**) mono- and (**D**) sub-nucleosomal average fragment frequencies at the TSS. Mono- and sub-nucleosome fragments were individually normalized after size selection to the effective genome size. Color code is shown in S2A. (**E**) Principal component (PC) analysis of whole genome nucleosome profiles. Mono-nucleosome profiles are displayed as triangle and sub-nucleosome profiles as circle. Color code is shown in S2A. (**F**) Correlation heatmap of whole genome nucleosome profiles. Pearson correlation coefficients are color coded and displayed in the heatmap.

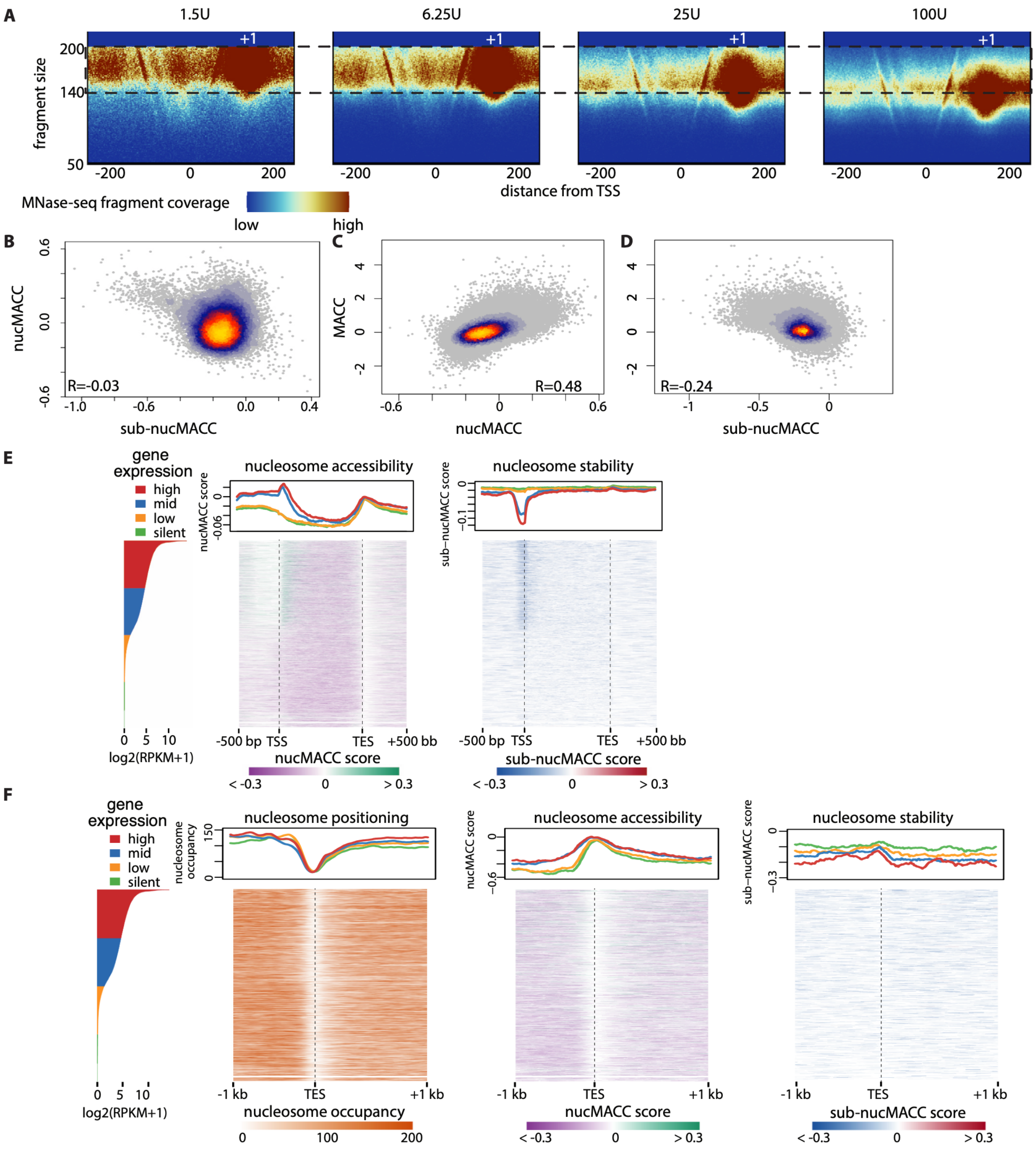

Fig. S3.

nucMACC scores at gene bodies and transcription end sites. (**A**) V-plots at TSS showing fragment length distribution across the different MNase concentrations. The +1 nucleosome directly downstream of the TSS is highlighted. Fragment sizes selected for mono-nucleosome analysis (140 - 200 bp) are highlighted by the dashed rectangle. (**B-D**) Density Scatterplot showing correlation between (**B**) sub-nucMACC and nucMACC scores, (**C-D**) original MACC score after (*31*) and either (**C**) nucMACC or (**D**) sub-nucMACC scores. Pearson correlation coefficients (R) are indicated. (**E-F**) Heatmaps sorted by gene expression showing nucleosome accessibility (nucMACC score), and stability (sub-nucMACC score) over scaled gene bodies (**E**) or at the transcription end site (TES) including nucleosome positioning (**F**). Genes were subdivided by gene expression quartiles, as indicated by the colors.

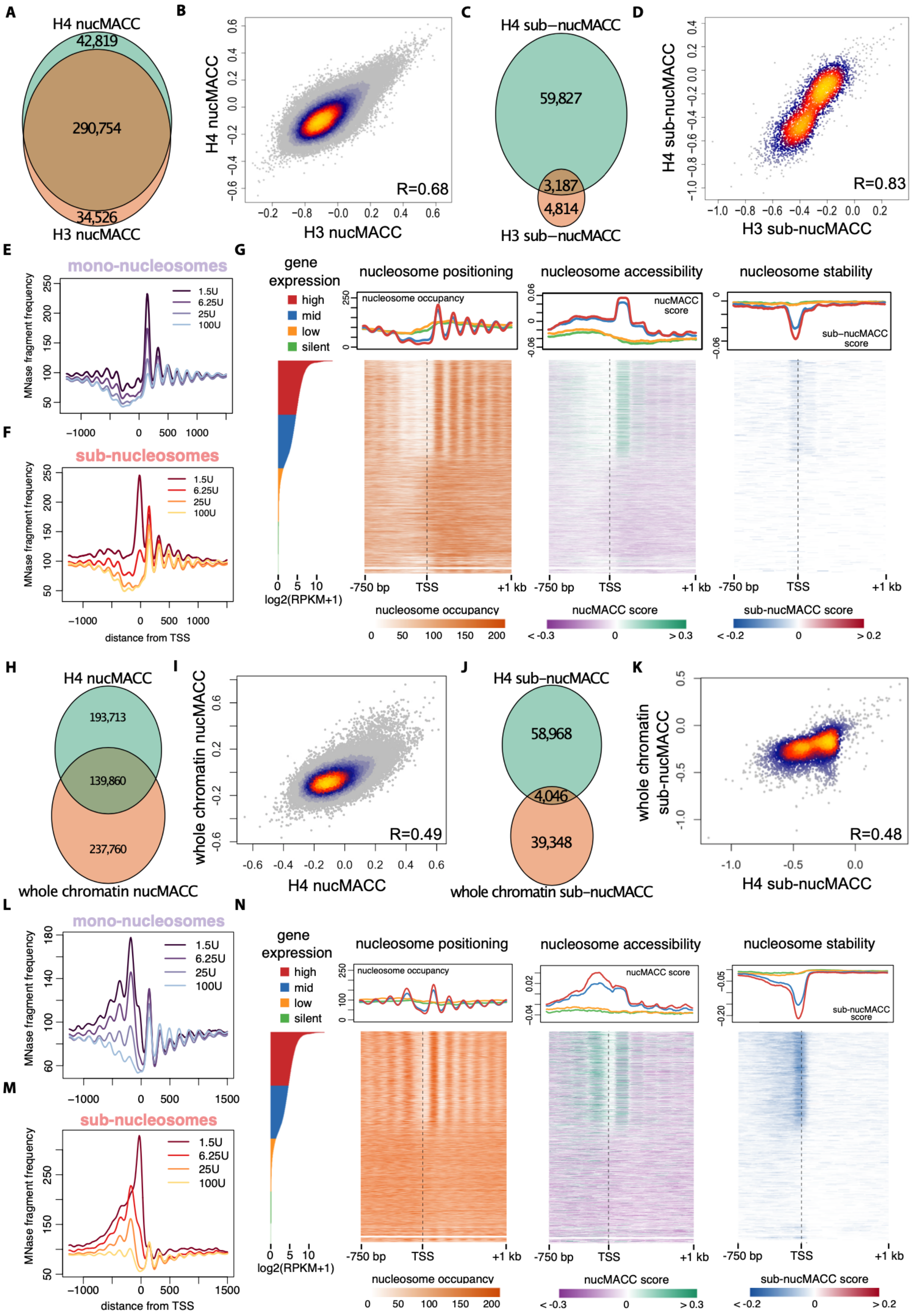

A
H4 nucMACC
42,819
290,754
34,526
H3 nucMACC
B
H4 nucMACC
H3 nucMACC
R=0.68
C
H4 sub−nucMACC
59,827
3,187
4,814
H3 sub−nucMACC
D
H4 sub-nucMACC
H3 sub-nucMACC
R=0.83
E
mono-nucleosomes
MNase fragment frequency
1.5U
6.25U
25U
100U
F
sub-nucleosomes
distance from TSS
G
gene expression
high
mid
low
silent
log2(RPKM+1)
nucleosome positioning
nucleosome occupancy
nucleosome accessibility
nucMACC score
nucleosome stability
sub-nucMACC score
-750 bp
TSS
+1 kb
H
H4 nucMACC
193,713
139,860
237,760
whole chromatin nucMACC
I
whole chromatin nucMACC
H4 nucMACC
R=0.49
J
H4 sub-nucMACC
58,968
4,046
39,348
whole chromatin sub-nucMACC
K
whole chromatin sub-nucMACC
H4 sub-nucMACC
R=0.48
L
mono-nucleosomes
M
sub-nucleosomes
N
gene expression
nucleosome positioning
nucleosome accessibility
nucleosome stability

Fig. S4.

Comparison of nucMACC scores with H3-ChIP or whole chromatin. (**A**) and (**C**) Venn diagrams showing the overlap of mono-nucleosome (**A**) or sub-nucleosome (**C**) positions determined using MNase combined with either H3 or H4 immunoprecipitations experiments in *D. melanogaster*. (**B**) and (**D**) Density scatterplot showing correlation of nucMACC (**B**) or sub-nucMACC (**D**) scores at overlapping positions between MNase combined with either H3 or H4 immunoprecipitations. (**E-F**) Average fragment frequencies at the TSS of MNase H3 data sets. (**G**) Heatmaps sorted by gene expression showing nucleosome positioning (left), accessibility (middle, nucMACC scores), and stability (right, sub-nucMACC scores) at TSS of MNase H3 data sets. (**H**) and (**J**) Venn diagrams showing the overlap of mono-nucleosome (**H**) or sub-nucleosome (**J**) positions determined using MNase either with H4 immunoprecipitation or without additional histone immunoprecipitation step (whole chromatin). (**I**) and (**K**) Density scatterplot showing Pearson correlation of nucMACC (**I**) or sub-nucMACC (**K**) scores at overlapping positions between MNase either with H4 immunoprecipitation or whole chromatin. (**L-M**) Average fragment frequencies at the TSS of MNase whole chromatin data sets. (**N**) Heatmaps sorted by gene expression showing nucleosome positioning (left), accessibility (middle, nucMACC scores), and stability (right, sub-nucMACC scores) at TSS of MNase whole chromatin data sets.

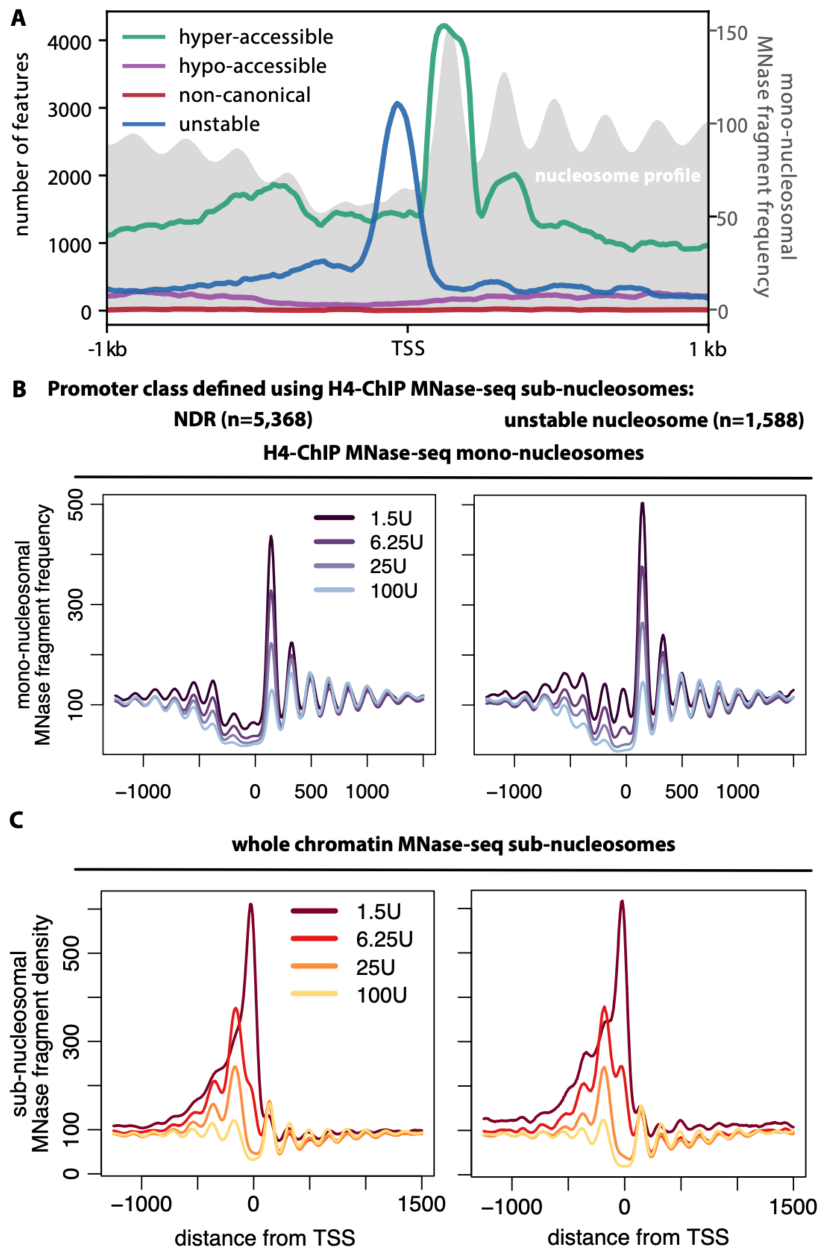

Fig. S5.

MNase data at promoter with or without unstable nucleosome. (**A**) Nucleosome feature distribution relative to the TSS. Background density displays the mono-nucleosome coverage profile of pooled samples. Average (**B**) mono-nucleosomal fragment frequencies of MNase H4-ChIP datasets and (**C**) sub-nucleosomal fragment frequencies of MNase whole chromatin datasets at the TSS of expressed genes either exhibiting a nucleosome depleted region (NDR) (n=5,368, left) or containing an unstable nucleosome (n=1,588, right) directly upstream of the TSS.

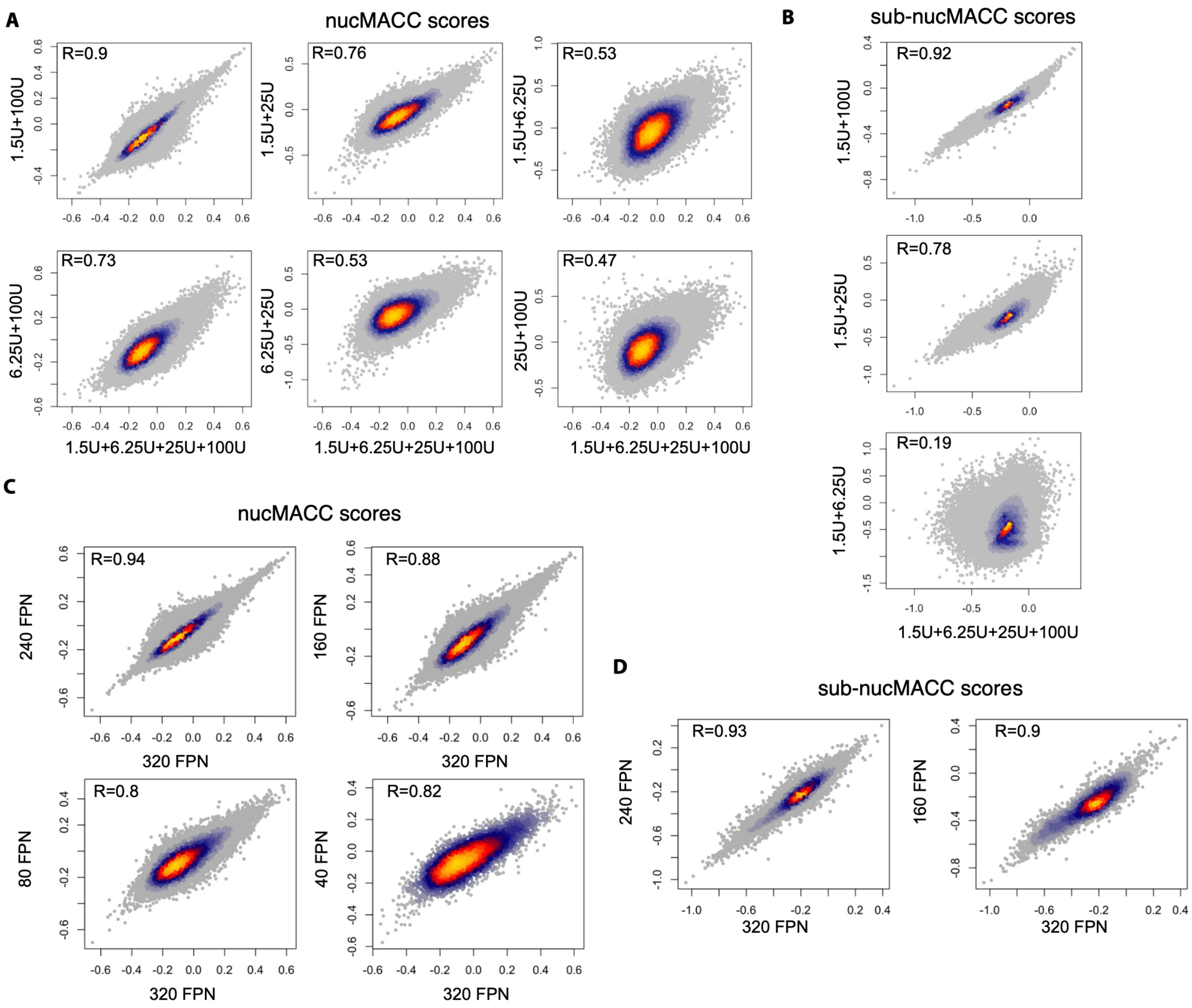

Fig. S6.

Impact of number of MNase conditions or sequencing depth on nucMACC scores. (**A-B**) Density scatterplots showing correlations of nucMACC (**A**) or sub-nucMACC (**B**) scores between all titrations and selected MNase titration pairs as indicated. (**C-D**) Density scatterplots showing correlations of nucMACC (**C**) or sub-nucMACC (**D**) scores between the total number of sequenced fragments (320 FPN) and subsampled fragments as indicated. FPN refers to the sequencing depth calculated as the number of fragments per nucleosome.

The Pearson correlation coefficient is indicated at the top.

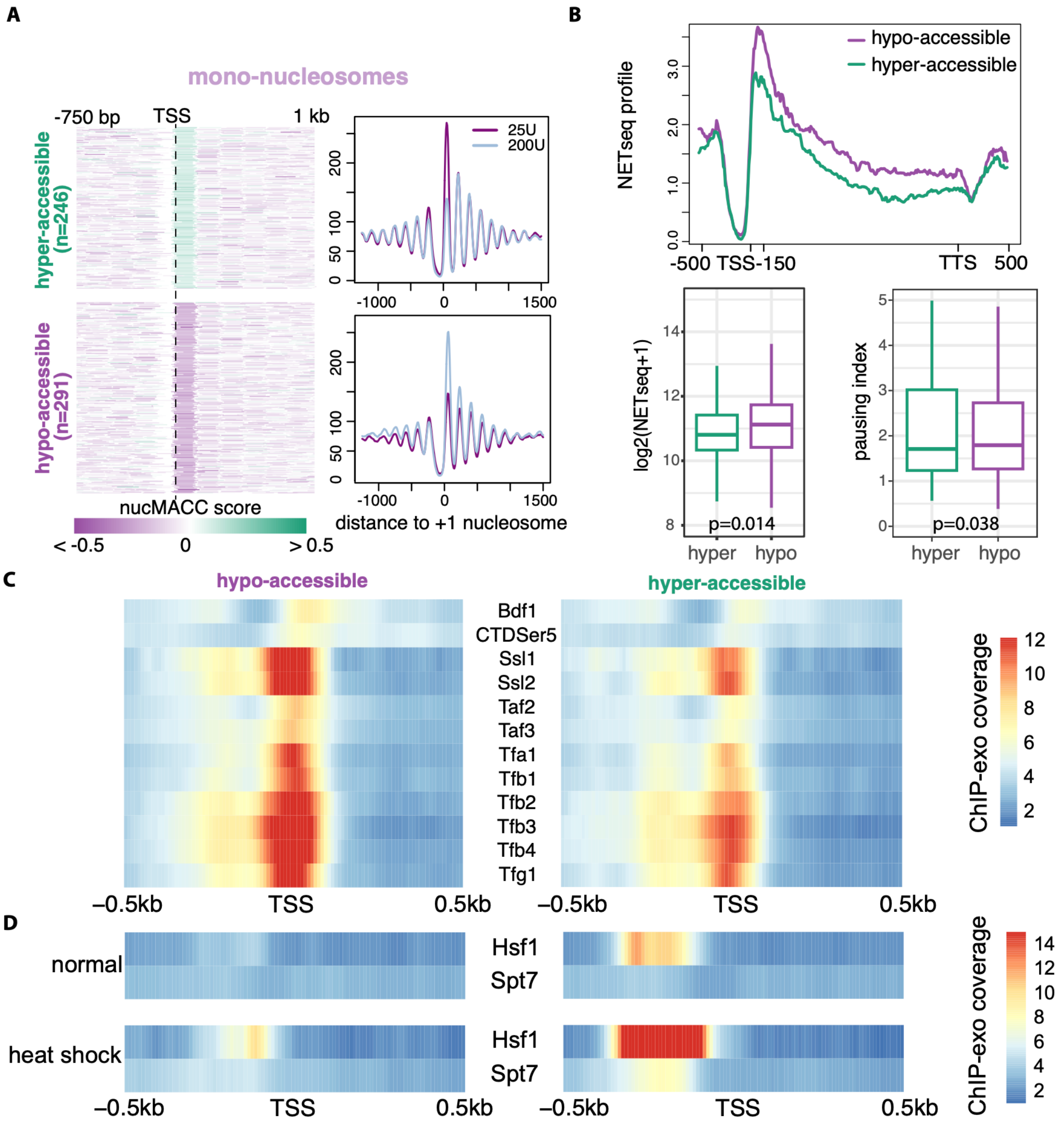

Fig. S7.

Hyper- and hypo-accessible nucleosomes in yeast. (**A**) Grouping of promoter types into promoter harboring a hyper- (n=246) or a hypo-accessible (n=291) +1 nucleosome in *S. cerevisiae*. Left panel: Heatmap showing the distribution of nuccMACC scores. Right panel: Average plot showing the mono-nucleosomal fragment frequencies of the different MNase conditions. (**B**) Gene expression analysis of the +1 nucleosome subgroups (hyper- in green and hypo-accessible in purple) using NETseq data. Top panel: Median NETseq profile showing nascent RNA abundance. Regions upstream of TSS, downstream of TTS and the first 150 bp downstream of TSS are unscaled. The remaining gene body is scaled to have the same length for each gene. Bottom panel: Boxplots showing the RNA Pol II initiation rate as calculated from the median NETseq signal over the +1 nucleosome (first 150 bp downstream of TSS) is shown on the left. The pausing index is shown on the right. Pausing index was calculated as the fold change of the NETseq signal in the first 150 bp downstream of the TSS versus the remaining gene body. (**C-D**) Heatmap illustrating occupancy of certain factors at hyper-/hypo-accessible +1 genes around the TSS. The color scale indicates the normalized coverage in ChIP-exo experiments (*56*).

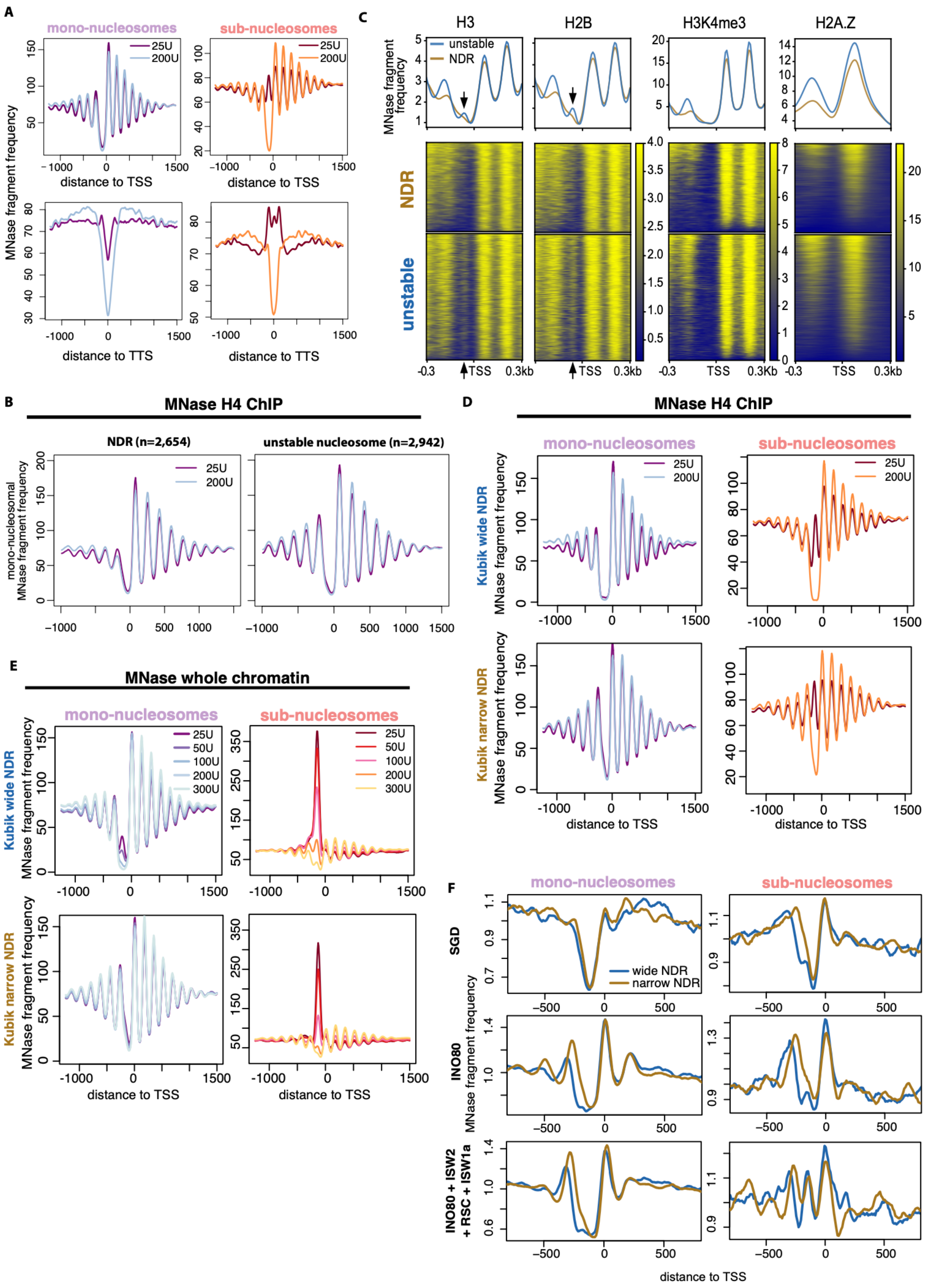

A
mono-nucleosomes
sub-nucleosomes
25U
200U
MNase fragment frequency
distance to TSS
distance to TTS
C
H3
H2B
H3K4me3
H2A.Z
unstable
NDR
MNase fragment frequency
TSS
-0.3
0.3kb
B
MNase H4 ChIP
NDR (n=2,654)
unstable nucleosome (n=2,942)
mono-nucleosomal MNase fragment frequency
D
MNase H4 ChIP
Kubik wide NDR
Kubik narrow NDR
E
MNase whole chromatin
50U
100U
300U
F
SGD
INO80
INO80 + ISW2 + RSC + ISW1a
wide NDR
narrow NDR

Fig. S8.

Unstable nucleosomes in yeast. (**A**) Average fragment frequencies of mono- (left) and sub-nucleosomal (right) fragments at the TSS (top) or TTS (bottom) in *S.cerevisiae*. (**B**) Average mono-nucleosomal fragment frequencies of MNase-H4-ChIP at the TSS of expressed genes either exhibiting a nucleosome depleted region (NDR) (n=2,654, left) or containing an unstable nucleosome (n=2,942, right) directly upstream of the TSS. (**C**) Meta-plot and heatmap showing fragment abundance at NDR and unstable nucleosome promoter. MNase-ChIP data sets without fragment size selection were used from (*56*). Arrows indicate the expected position of unstable nucleosomes. (**D-E**) Average fragment frequencies of MNase-H4-ChIP (**D**) or MNase whole chromatin (**E**) data sets at the TSS subgrouped by NDR width after (*37*). (**F**) MNase fragment density of *in vitro* reconstituted nucleosome arrays at unstable narrow or wide NDRs. MNase fragments were size selected into mono-nucleosomal (140 bp – 200 bp; left panel) and sub-nucleosomal fragments (< 140 bp; right panel). Nucleosomes were assembled onto DNA by salt gradient dialysis (SGD) and purified remodeler were added as indicated (*57*).

Data S1. (separate file)
Overview of MNase data sets analyzed in this study using the nucMACC pipeline.

Data S2. (separate file)
Results of nucMACC analysis in *D.melanogaster*.

Data S3. (separate file)
Results of nucMACC analysis in *S.cerevisiae*.

Data S4. (separate file)
List of human genes with unstable chromatin particles in their promoter upon shH2A.Z.
